# Lymphocytic Thyroiditis – is cytological grading significant? A correlation of grades with clinical, biochemical, ltrasonographic and radionuclide parameters

**DOI:** 10.1186/1742-6413-4-10

**Published:** 2007-04-30

**Authors:** Alka Bhatia, Arvind Rajwanshi, Radharaman J Dash, Bhagwant R Mittal, Akshay K saxena

**Affiliations:** 1 Department of Cytology, Postgraduate Institute of Medical Education and Research, Chandigarh, India; 2 Department of Endocrinology, Postgraduate Institute of Medical Education and Research, Chandigarh, India; 3 Department of Nuclear medicine, Postgraduate Institute of Medical Education and Research, Chandigarh, India; 4 Department of Radiodiagnosis, Postgraduate Institute of Medical Education and Research, Chandigarh, India

## Abstract

**Background:**

Clinical, biochemical, ultrasonographic, radionuclide and cytomorphological observations in Lymphocytic thyroiditis (LT), to define the cytological grading criteria on smears and correlation of grades with above parameters.

**Methods:**

This prospective study was conducted on 76 patients attending the Fine needle aspiration cytology clinic of a tertiary care institute in North India. The various parameters like patients' clinical presentation, thyroid antimicrosomal antibodies, hormonal profiles, radionuclide thyroid scan and thyroid ultrasound were studied. Fine needle aspiration of thyroid gland and grading of thyroiditis was done on smears. The grades were correlated with above parameters and the correlation indices were evaluated statistically.

**Results:**

Most of the patients were females (70, 92.11%) who presented with a diffuse goiter (68, 89.47%). Hypothyroid features (56, 73.68%) and elevated TSH (75, 98.68%) were common, but radioiodide uptake was low or normal in majority of patients. Thyroid antimicrosomal antibody was elevated in 46/70 (65.71%) patients. Cytomorphology in fine needle aspirates was diagnostic of lymphocytic thyroiditis in 75 (98.68%) patients. Most of them had grade I/II disease by cytology. No correlation was observed between grades of cytomorphology and clinical, biochemical, ultrasonographic and radionuclide parameters.

**Conclusion:**

Despite the availability of several tests for diagnosis of LT, FNAC remains the gold standard. The grades of thyroiditis at cytology however do not correlate with clinical, biochemical, radionuclide and ultrasonographic parameters.

## Background

The first report of chronic thyroiditis, *struma lymphomatosa *was described by Hakaru Hashimoto in 1912, which bears his name. Although Hashimoto's thyroiditis is sometimes referred to goitrous thyroiditis, it may usually be considered, a synonym of chronic lymphocytic thyroiditis or autoimmune thyroiditis including atrophic and non-goitrous thyroiditis [1]. Hashimoto's thyroiditis is characterized by Hurthle cell change and an increased number of mature and transformed lymphocytes impinging on follicular cells [2]. The autoimmune process is believed to begin with the activation of CD4+ T-cells which initiate the recruitment of auto reactive B-cells that secrete variety of thyroid antibodies, the important ones being antithyroglobulin, thyroid microsomal (TMA) and thyroid stimulating hormone stimulation blocking antibodies [3,4]. Out of these the TMA titers correlate best with the degree of thyroidal lymphocytic infiltration [5]. Besides antibody titers, thyroid hormonal profile and radioactive iodide uptake (RAIU) help in assessing the functional status, but the information obtained from thyroid function tests is "soft" as the secretion of thyroid stimulating hormone is influenced by many factors [1]. Thyroid hypoechogenicity on ultrasound (USG) correlates well with lymphocytic infiltration [6,7] and levels of circulating antibodies [8].

Fine needle aspiration cytology (FNAC) of thyroid provides a safe and accurate method for diagnosis of the condition. Cytological grading of thyroiditis on smears, based on a set of predefined criteria has not been carried out in the past. This study was one such, aimed at grading the thyroiditis and correlating the grades with clinical, biochemical, ultrasonographic, radionuclide parameters and serum TMA levels.

## Methods

Seventy six consecutive patients attending our FNAC clinic from January 2002 to December 2004 and clinically presumed to have chronic lymphocytic/autoimmune thyroiditis were recruited for this study. A written consent was obtained from each patient for inclusion in the study. The patients had estimation of T3, T4, TSH, thyroid microsomal antibodies (using serodia AMC Kit), ^131^I-thyroid uptake and high resolution thyroid USG (using 7–12 MHz Broad band linear transducer on HDI 5000 of ATL, Japan) to locate macronodules (focal lesions ≥ 5 mm in diameter).

Fine needle aspiration of the thyroid was performed from several locations. USG guidance was not used for the procedure. Smears were prepared and stained with May Grunwald Giemsa, Papanicolaou/Haematoxylin and eosin. In case the material obtained was not satisfactory a repeat aspiration was done but not more than 2–4 aspirations were tried on each patient. The smears were seen by two independent cytologists. Qualitative criteria used for cytologic diagnosis were lymphocytes and plasma cells infiltrating the thyroid follicles and increased number of lymphocytes in the background with or without lymphoid follicles, Hurthle cell change, multinucleated giant cells, epithelioid cell clusters, anisonucleosis or interlobular fibrosis that is the presence of fibrous tissue or scattered fibroblasts in the aspirate. Quantitation of thyroiditis was done by a cytological grading system based on number of lymphocytes infiltrating the gland, the degree of destruction caused (relative proportion of inflammatory and follicular epithelial cells) and presence of associated features like Hurthle cell change, giant cells, anisonucleosis etc (Table 1). Mann Whitney and chi-square tests were used for statistical correlation and p-value of <0.05 was considered significant.

**Table 1:** Grading of thyroiditis on cytological material

**Grade**	**Morphological features**	**Percentage in our study**
Grade 0	No lymphoid cells.	0
Grade I (Mild)	Few lymphoid cells infiltrating the follicles/increased number of lymphocytes in the background.	38.67%
Grade II (Moderate)	Moderate lymphocytic infiltration or mild lymphocytic infiltration with Hurthle cell change/giant cells/anisonucleosis.	44%
Grade III (Severe)	Florid lymphocytic inflammation with germinal center formation, very few follicular cells left.	17.33%

## Results

Out of 76 patients 70 (92.11%) were females and 6 (7.89%) were males. Their age ranged from 6 to 60 years (mean age females, 34.2 years, and males 37.2 years) with majority in 3^rd ^and 4^th ^decades. All but one patient had lymphocytic thyroiditis on FNAC.

On examination while performing FNAC, 68 (89.47%) had diffuse goiter, 2 (2.63%) had nodular goiter and 6 (7.9%) had no goiter. Fifty six (73.68%) patients experienced hypothyroid symptoms, 1 (1.32%) had toxic features and 19 (25%) were euthyroid. Seventy five patients (98.68%) had low or normal T3, T4 with high TSH, while one had normal T3, T4 and TSH. TMA titers were also not elevated in this patient. However grade I thyroiditis was noted on FNAC and USG showed a solitary nodule. The scintigraphy was performed in 65 patients and it revealed low or normal RAIU in 53 (81.54%) and a high value in 12 (18.46%) patients.

The serum TMA titers were determined in 70 cases. The values were elevated in 46/70 (65.71%) and normal in 24 (34.29%). The sensitivity of TMA when compared to FNAC was 62.6%. USG was carried out in 48 cases. The features observed were hypoechogenic goiter (2, 4.16%), micronodules <5 mm (35, 72.92%), dominant nodules ≥5 mm (12, 25%) and echogenic septations (24, 50%). The dominant nodule was hyperechoic in one case and hypoechoic in the rest. Sonographic goiter (Volume more than 18 ml in females and more than 25 ml in males) was seen in 18 (37.5%) patients.

**Figure 1:**
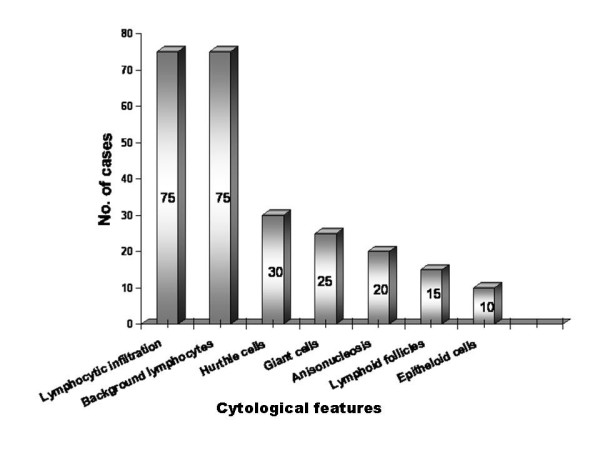
Cytomorphological features in lymphocytic thyroiditis.

**Figure 2:**
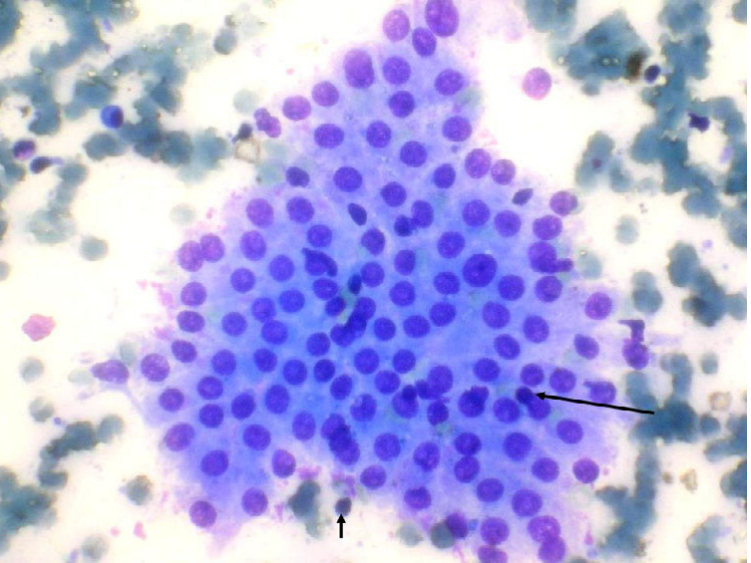
Grade I thyroiditis with mild lymphocytic inflammatory infiltrate (arrows) (May Grünwald Giemsa).

**Figure 3:**
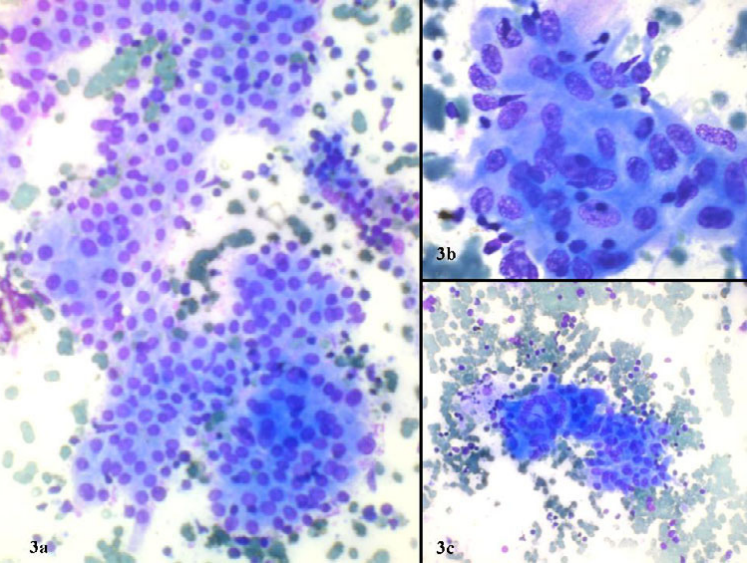
Grade II thyroiditis with a) moderate lymphocytic inflammation b) epithelioid cell granuloma c) multinucleated giant cell (May Grünwald Giemsa).

**Figure 4:**
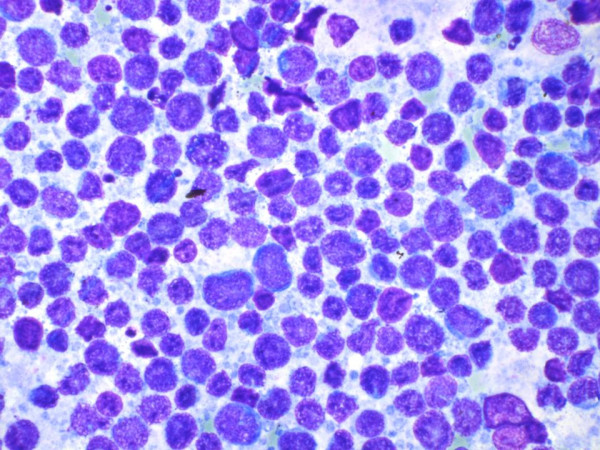
Grade III thyroiditis, marked inflammation, and germinal center formation as in a reactive node. Note polymorphic population of lymphoid cells in contrast to a lymphoma (May Grünwald Giemsa).

The FNAC was performed from multiple sites and out of 76 patients 75 (98.68%) had lymphocytic thyroiditis. The morphological features observed are shown in figure 1. In one patient the FNAC failed to yield sufficient material for diagnosis and also this patient did not return to clinic for a repeat aspiration. Grading of thyroiditis was done as per the criteria mentioned (Table 1). Twenty nine (38.67%) patients had mild lymphocytic infiltration of the gland and were graded as Grade I thyroiditis (figure 2). Thirty three patients (44%) had grade II disease characterized by moderate degree of infiltrate with evidence of follicular destruction, Hurthle cell change, giant cells etc (figure 3). Grade III thyroiditis was noted in 13 (17.33%) patients who showed dense infiltrates with germinal centers and with very few follicular cells left (figure 4). The polymorphic population of cells with lymphocytes, immunoblasts and plasma cells helped in distinguishing these cases from Non-Hodgkin's lymphoma. Table 2 summarizes the results and shows comparison with some of the other studies.

**Table 2:** Lymphocytic thyroiditis – Contrasted and compared with some of the previous studies

**Author**	**No. of Pts**	**Age (yrs)**	**Sex**	**Cytologic diagnosis**	**Clinical Presentation**		**Hormonal changes (%)**	**TMA (%)**	**RAIU**	**USG**	**Cytological grading**
										
					Diffuse	Nodular					
Kumar et al^10^	55	7–45	55F	All LT	81.8%	18.2%	72	83.6	-	-	Mild = 61.90% Mod. to Heavy = 38.1%
Nguyen et al^11^	146	15–70	139F 7M	All LT	71.92%	28.08%	35	-	-	-	-
Kini et al^22^	87	-	-	60LT (17on biopsy)	78.16%	-	49.33	61.64	↓ in nodules	-	-
Friedman et al^12^	40	18–71	33F 7M	All LT	5%	80%	7.5	75	↑ = 2.5% ↓ = 50% N = 47.5%	-	-
Jayaram et al^2^	40	40–50	40F	37LT	-	-	45	57.5	n = 10 All thyrotoxic ↑ = 100%	n = 30 Hypo- anechoic	n = 37 I = 13.51% II = 62.16% III = 24.32%
Bhatia et al	76	6–60	70F 6M	75 LT	89.47%	2.63%	98.68	n = 70 65.71	n = 65 ↑ = 18.46% ↓ = 30.77% N = 50.77%	n = 48 micro nodules	n = 75 Grade I = 38.67% Grade II = 44% Grade III = 17.33%

LT = Lymphocytic thyroiditis, F = Females, M = Males, TMA = Thyroid microsomal antibodies, RAIU = Radioactive iodide uptake, USG = Ultrasonography, ↓ = decreased, ↑ = increased, N = Normal Pts = Patients

Of the seventy six patients, data on all parameters was available in 48 cases (63.16%) only. Statistical correlation of grades of thyroiditis with all the above parameters was carried out in them but it was not found to be significant (p-value >0.1).

## Discussion

Chronic lymphocytic thyroiditis is an autoimmune entity where in the thyroid follicles are rapidly destroyed. The cytological markers include lymphocytic infiltration of the interfollicular space, invasion of follicles by the lymphocytes giving a fire-flare appearance characterized by eosinophilic vacuolated cytoplasm and later, total destruction of follicles. In the long run, the follicular architecture is totally destroyed and replaced by fibrosis. The active phase of disease is transient with clinical manifestations of thyrotoxicosis while the evolution phase and destructive phase manifest with subclinical or overt hypothyroidism. The present study was designed to correlate the cytological grades of thyroiditis with rest of the parameters and to define the grading criteria for thyroiditis on cytological material.

Our patients were mainly young females. This is in contrast to a previous study [9] from United Kingdom in which the patients were mainly older women with mean age at diagnosis being 59 years. This disparity may be due to occurrence of LT in young patients in iodine deficient areas such as ours, while it occurs in older individuals in iodine sufficient areas [2,10]. Most of our patients had symptoms of hypothyroidism with a diffuse goiter although nodular presentation was also noted in few cases. Previous authors have also documented nodular presentation in LT which might mimic malignancy clinically [11]. Friedman et al [12] found nodular presentation in as many as 80% of their patients. The nodules represent early stage of the disease when the clinical and hormonal changes are not established. In our study the incidence of nodularity was low as the patients had either sub clinical or clinical disease, at the time of presentation. TSH was elevated in all but one case with either decreased or normal T3, T4. The normal T3, T4 levels in the presence of elevated TSH indicate sub clinical hypothyroidism (SCH). The incidence of SCH was higher in our study compared to a study by Bagchi et al [13] who found subclinical disease in 8–17% of their subjects. Higher incidence of overt or subclinical hypothyroidism in our subjects is understandable as they were from a clinic population where individuals with subtle or significant symptoms are expected to seek medical advice. One of our patients was hyperthyroid with high T3, TSH and uptake levels and increased TMA titers. Her thyroid aspirate showed grade III thyroiditis. This phenomenon known as thyrotoxicosis is due to acute aggravation of thyroid autoimmunity induced destruction of follicles.

The RAIU values were normal or low in majority of the patients as was seen in previous studies [12]. The low uptake values indicate the destruction of gland by the autoimmune process. The raised RAIU in some may be due to iodine deficiency.

Serum TMA titers were elevated in more than half of our patients in contrast to published reports where the serum TMA titers were elevated in up to 95% patients [14]. Such variations are explained by chance inclusion of patients early in the course of disease as intrathyroidal immune destruction occurs much earlier to serological evidence and inclusion of non-immune LT in the patient group [15].

In this study the sonographic goiter was found in less number of patients as compared to those with clinical goiter. This discrepancy may be due to lack of reference values in radiology for Indian population. Also it has been shown that thyroid volume varies significantly with factors like age, sex, height, weight and place of living [16,17]. Hence the western data cannot be extrapolated to Indian population and may be the source of lower prevalence in our study. Micronodularity was the most frequently observed feature and showed a high positive predictive value which is in keeping with the observations in previous studies [18]. Fifty percent patients showed septations on USG indicating fibrosis and hence disease of some duration. The low prevalence (4.16%) of diffuse hypoechogenic enlargement of thyroid gland in comparison to previous studies (18.5–95%) [8,19] may also be due to relatively small number of patients in our study group. The USG helps not only in diagnosis of the condition but also in selecting the patients with suspicious nodules for work up for malignancy [20]. In the present study no neoplastic lesion was found amongst the cases studied.

The diagnostic accuracy of fine needle aspiration cytology was high, multiple aspirations being helpful in almost all cases. The usefulness of increased number of aspirations has been stressed by Hamburger et al [21] who found that as the number of aspirations increased, false negative results decreased. It has been emphasized in the past that in equivocal cases antibody testing is helpful, but if negative, a repeat FNA becomes the ideal choice [10]. Only in one case with high serum TMA and TSH levels was the FNAC non-contributory due to inadequate material. A repeat sampling could not be performed in this case.

Grading of thyroiditis has been carried out on histological specimens in the past based upon number of foci of lymphocytes per standard representative section [23]. On the other hand, grading on cytology smears has been done by only a few workers. In this study for the first time grading was carried out on FNAC smears using a set of predefined criteria. The grading using these criteria was found to be quite consistent and a high concordance rate was noted amongst the two observers. The grades were statistically correlated with clinical, biochemical, radionuclide, ultrasonographic features and serum TMA levels. It was observed that many of the patients with grade III disease had features of hypothyroidism and an altered hormonal profile however statistical correlation between the grades and above parameters was not significant. Kumar et al [10] carried out correlation of severity of lymphocytic infiltration on smears with functional and antibody status however, in their study also no significant correlation was found. This may be due to the fact that grading on FNAC smears is also affected by other factors like dilution by blood, technique of the FNAC and the number of aspirations used. Furthermore, the aspirates are obtained from very tiny portion of thyroid gland and may not at times represent the pathology in entirety.

Therefore to conclude, lymphocytic infiltration of thyroid follicles is pathognomonic of lymphocytic thyroiditis and hence FNAC remains the gold standard for diagnosis. When graded into mild, moderate and severe the grades of thyroiditis correlate poorly with clinical, biochemical, ultrasonographic and radionuclide features and serum TMA levels.

## Competing interests

The author(s) declare that they have no competing interests.

## Authors' contributions

AB: Acquisition, analysis and interpretation of data and drafting the manuscript

AR: Conception and design of the study, interpretation of data

RJD, AS and BRM: Acquisition, analysis and interpretation of data

All the authors read and approved the final manuscript
